# Exploring health disparities in congenital CMV (cCMV): a study in a Somali-American community to assess awareness of cCMV and facilitate understanding of universal cCMV screening

**DOI:** 10.1007/s44155-024-00070-8

**Published:** 2024-03-26

**Authors:** Khadra Hussein, Ryan Shanley, Mark R. Schleiss

**Affiliations:** 1 University of Minnesota Medical School, 420 Delaware Street SE, Minneapolis, MN 55454, USA.; 2 Biostatistics Core, University of Minnesota Clinical and Translational Science Institute, 717 Delaware Street SE, Minneapolis, MN 55414, USA.; 3 Division of Pediatric Infectious Diseases, University of Minnesota, 2001 6th Street SE, Minneapolis, MN 55455, USA.

**Keywords:** Black/African–American/Somali–American, Cytomegalovirus (CMV), Congenital CMV infection, Community-based

## Abstract

**Background:**

Congenital cytomegalovirus (cCMV) disproportionately impacts black and multiracial infants. While there have been strides made to address this health disparity, strategies to increase awareness and knowledge of cCMV have not been investigated in a Somali community.

**Methods:**

Two survey study strategies (in-person and online), consisting of a pre-survey test, educational intervention, and a post-survey, were designed to gauge knowledge and perceptions about cCMV among Somali women aged 18 to 40 years old.

**Results:**

96 respondents partook in the online module, and 15 in the in-person event. On recruitment, < 45% of women were aware of cCMV. Following the pre-intervention survey, educational modules were conducted, and the survey repeated. For statistical comparisons, a point was assigned for each correct survey query, and the mean of correct responses tabulated for pre- and post-surveys. In the online intervention, mean scores changed from 55 to 87% (paired *t*-test, p = 0.001), whereas in the in-person intervention, mean scores changed from 65 to 87% (paired *t*-test, p = 0.007), demonstrating enhanced cCMV awareness upon completion of both interventions. Using multiple linear regression, the expected post-test score was 2% (95% CI [− 8%, 12%]) higher for the online module compared to the in-person module, adjusting for pre-test score.

**Conclusion:**

Both interventions were successful in enhancing knowledge about cCMV in this population, although there was no evidence either intervention was substantially better than the other. Educational efforts will be critical in enhancing the trust required to facilitate diagnostic evaluation and treatment of newborns identified with cCMV in this high-risk population.

## 1 Introduction

Congenital cytomegalovirus (cCMV) is a major public health concern. Infection with this viral pathogen in a newborn represents the most common infectious cause of neurodevelopmental disability in the US [1], and probably globally [2, 3]. Of particular concern is the potential for sensorineural hearing loss (SNHL), which occurs in 12–20% of all children with cCMV [4–7]. It is estimated that 21% of all hearing loss at birth is caused by cCMV, and that by 4 years of age, 25% of pediatric SNHL is caused by this infection [8, 9]. Paradoxically, cCMV prevalence is directly proportional to the seroprevalence of CMV antibodies in the maternal population being examined [10–13]. Since CMV seroprevalence is higher in black and African-American communities [14–17], cCMV infections reflect significant racial and socioeconomic disparities in the US, as confirmed in the recent landmark CHIMES study [18, 19]. Analyses of death certificate data demonstrate that Native American and African-American infants have an increased risk of cCMV-related mortality compared to white infants [20].

One population of particular interest in the consideration of racial disparities in the US is the Somali-American population. At > 50,000, Minnesota has the largest population of people who report Somali ancestry of any state in the US. Somali immigrants have faced many challenges related to discrimination, disparities, and lack of trust in US care providers [21], although these deficiencies are amenable to educational interventions [22]. Distrust among member of the Minnesota Somali-American community was embodied by a major measles outbreak that took place in 2017 [23], driven by disproven links between the mumps-measles-rubella (MMR) vaccine and autism-spectrum disorders [24] which impaired vaccine compliance [25]. Only 41% of parents expressing willingness to immunize their future children with MMR, even in spite of the measles outbreak in their community [26].

Culturally sensitive educational programs about important health concerns have been demonstrated to increase knowledge and a sense of trust in Minnesota’s Somali-American community for diverse issues such as breast and cervical cancer screening [27], type 1 diabetes [28], and genetic counseling [29]. Driven by recent successes in optimizing newborn screening methodologies for cCMV infection [30], the Minnesota Department of Health (MDH) recently announced that it would commence universal screening for all newborns for cCMV, beginning in 2023. However, knowledge and awareness of cCMV is low across all communities, racial and ethnic groups, and socioeconomic strata [31–34], even among women who are healthcare providers [35]. Implementation of universal cCMV screening will likely include an extensive evaluation of the infected newborn, creating a risk for disparities in care [36]. It is therefore important to consider possible resistance to these recommendations due to the extensive medical evaluation for an infant required following a positive cCMV newborn screen. Therefore, these studies were undertaken to gauge knowledge and awareness of cCMV in the Somali-American community in Minnesota, and to examine the impact of an educational intervention meant to increase knowledge among young women about the importance of cCMV, toward the long-term goal of ensuring acceptance of cCMV screening in this population.

## 2 Methods

### 2.1 Human subjects

The study protocol, informational sheets, and questionnaires were reviewed by the University of Minnesota (UMN) Institutional Review Board (IRB; STUDY00016509). The study was found to meet the UMN criteria for IRB exemption.

### 2.2 Data collection

**Fig. 1:**
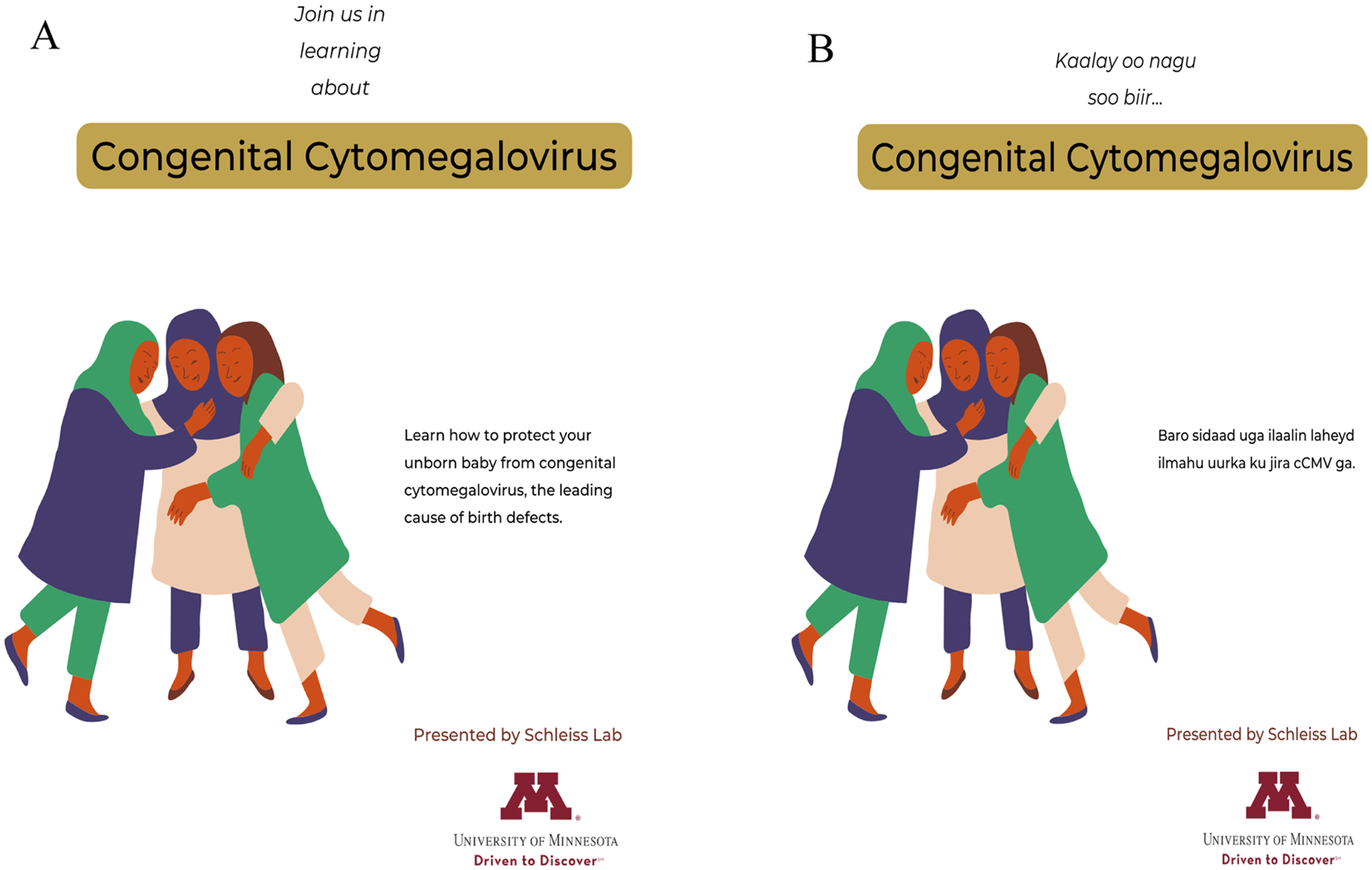
Flyers used in recruitment for this study, prepared both in English (**A**) and Somali (**B**)

To establish trust and leverage support for the study, recruitment began in early April 2022. Partnership with head Imaam (spiritual leader) was established at a mosque in Burnsville, MN. Announcements about the study took place during the congregation at the Friday prayers. Additionally, advertisements in the form of flyers were shared and posted on the mosque boards and on social media (Fig. 1). Information about the in-person event was also shared on the Somali TV channel (https://www.facebook.com/SOMTVMN/videos_by).

The study was conducted in two cohorts: both an in-person, face-to-face educational event, and a web-based survey. In both, women self-selected to either participate in the online survey or the in-person event. The inclusion criteria were: (1) Somali woman; and (2) of a reproductive age ranging from 18 and 40 years. The purpose of the survey was two-fold: (1) to evaluate the awareness of cCMV in the Somali community using a pre- and post-test survey; and (2) to increase women’s knowledge of cCMV transmission, prevention, and available treatments, through administration of the educational modules.

For the web-based survey, potential participants found out about the study through a QR code shared on social media, and these individuals therefore had some minimal essential information about cCMV provided to them in advance of the survey. Similarly, the in-person participants recruited at the mosque had received basic information about cCMV through verbal announcements and posting of recruitment flyers. For those who pursued participation, the following information was provided: *“Cytomegalovirus is the leading cause of congenital abnormalities in newborns. The data findings will be used to help identify initiatives tailored towards Somali women and their families. All the answers you provide will be confidential and will not be shared with anyone other than members of the team. Completion of this survey is voluntary, but we hope you will agree to answer the questions as your views are important. Please note, to receive the gift card, it is vital you complete the entire form. This includes watching the full duration of the presentation. If you have any questions, please reach out to me at* huss0248@umn.edu.*”*

**Fig. 2:**
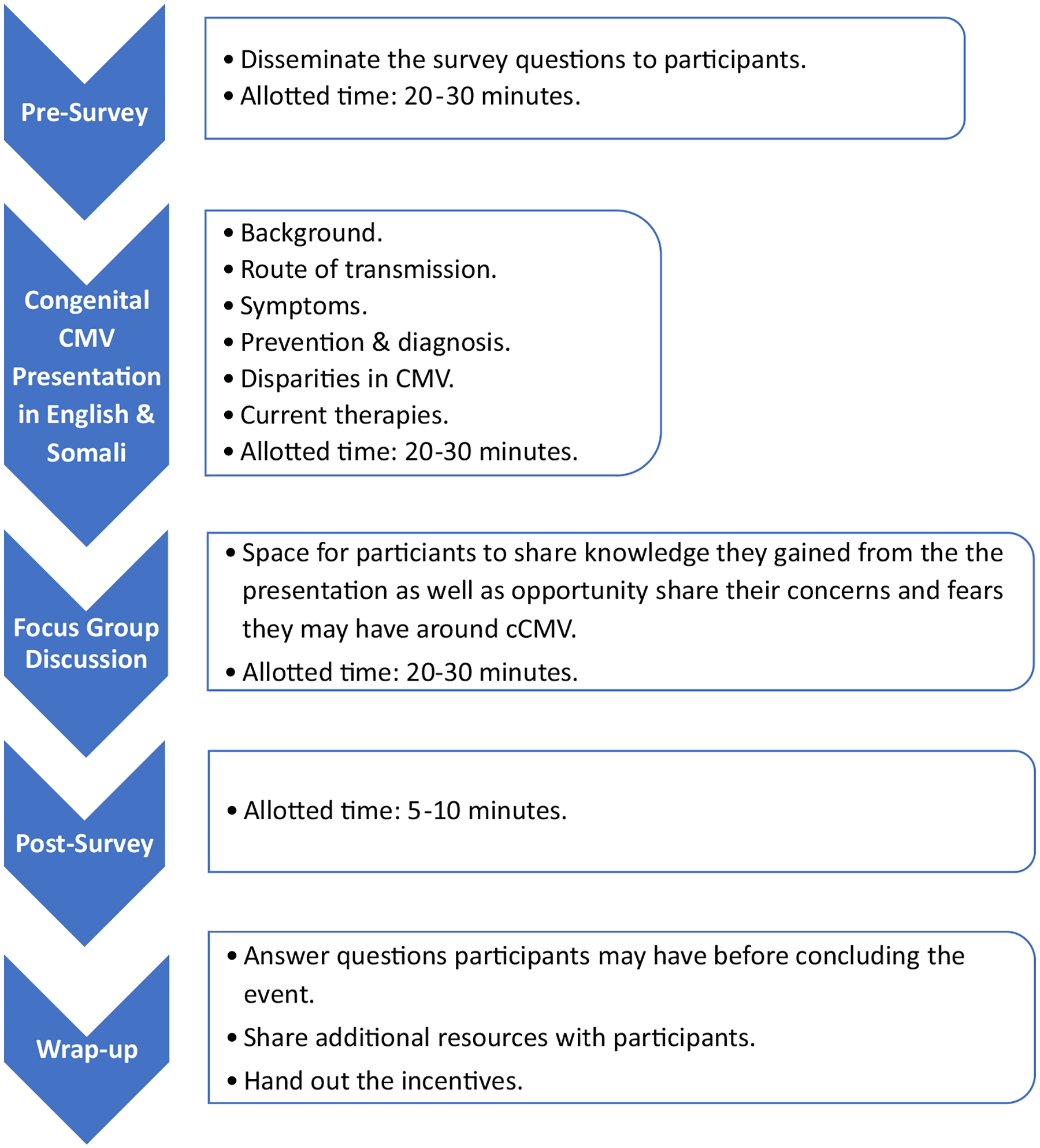
Agenda utilized for the in-person educational event

For the in-person survey, as noted, verbal recruitment at the participating mosque was used to solicit interest in the study. The recruitment flyer is shown in Fig. 1. The handout summarizing the in-person event agenda used for the educational program at the mosque is shown in Fig. 2.

**Fig. 3:**
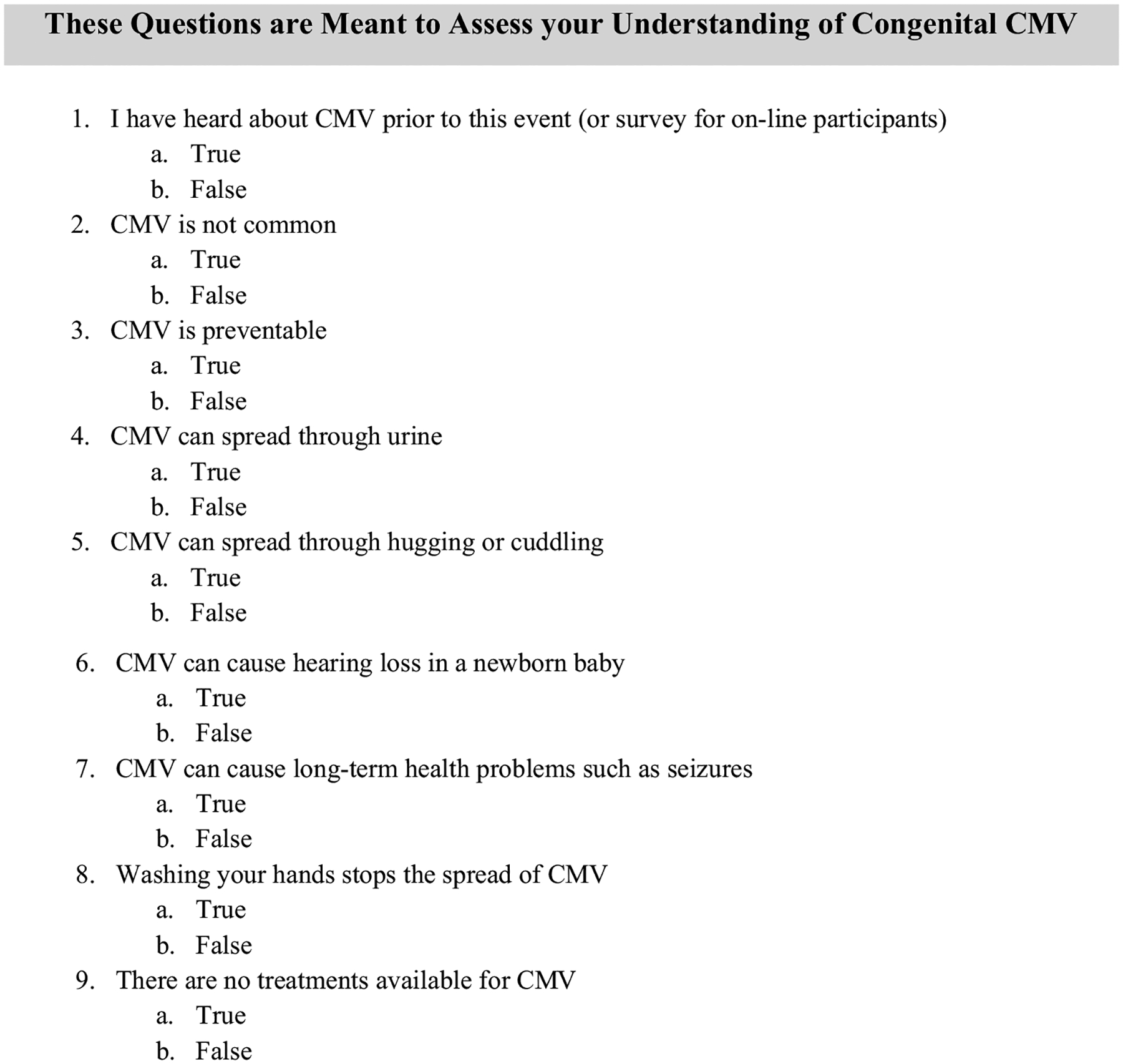
Survey statements disseminated in both types of sessions (internet-based and in-person)

In both cohorts, the study was conducted in four steps: (1) participants identified their socio-demographic information; (2) participants answered statements assessing their knowledge and attitude of cCMV; (3) participants partook in the intervention; and (4) participants answered the same statements as they did in step 2. In the in-person face-to-face educational event, a discussion followed the intervention, where participants had the opportunity to ask questions. To accurately capture women’s knowledge and attitudes around cCMV and to gauge how effective the intervention was, identical pre- and post- survey statements were disseminated in both sessions. The only exception occurred in the post-survey questionnaire in both sessions, where a pre-survey statement assessing women’s familiarity with cCMV was not repeated in the post-survey (since all participants, by definition, had heard about cCMV at that point). All survey statements were queried in a true-or-false format (Fig. 3). The statements in the questionnaire were related to the following categories: (1) prevention of cCMV; (2) modes of transmission; and (3) diagnosis and treatment of cCMV.

### 2.3 Statistical analyses

Data was analyzed using a combination of Excel and Statistical Product and Service Solutions (SPSS) software. Descriptive analysis statistics such as frequencies were identified and calculated. Additionally, inferential statistics were conducted using paired sample *t*-tests, multiple linear regression, and a chi square test. A paired sample *t*-test was utilized to determine the effectiveness of the intervention and a multiple linear regression to identify which method (the in-person vs. the web-based method) was more effective in teaching women about cCMV. Additionally, chi- square tests were used to explore the relationship between socio-demographic characteristics and women’s awareness of cCMV prior to the study.

## 3 Results

There were 96 responses for the web-based survey and 15 responses for the in-person survey, for a total of 111 responses. Details for the web-based and in-person surveys are summarized below.

### 3.1 Demographics

Of the 96 respondents that filled the web-based questionnaire, 37% were between the ages of 28 and 34 while 27% were between 21 and 27 years. Most of the participants obtained a high school education or less and earned between $50,000 and $99,000. Of the 96 respondents, 61 participants, or 64%, said they had previously been pregnant. For the in-person questionnaire, 48% of the 15 participants were over 35 years old. Additionally, most of the participants had received a high school education or less and earned between $20,000 and $49,000. In addition, there were 7 respondents or 47% of the participants who indicated that they were previously pregnant.

### 3.2 Survey data: impact of intervention and relationship to pre-survey familiarity with cCMV

Women answered nine statements, with the first statement in the pre-survey not being repeated in the post-survey (and hence, not utilized in comparing the pre/post survey scores). In this statement, women were asked if they had heard about cCMV prior to engaging in this study. In the web-based survey, 44.8% of the women indicated they had heard of cCMV, and 40% in the in-person session indicated they had heard of cCMV prior to the event (Table 1).

To elucidate the effectiveness of the intervention for the eight remaining pre- and post-education survey queries, a mean of the correct responses was tabulated. A point was given for each correct survey statement selected, and then the mean of correct responses was tabulated for the pre/post surveys. In the web-based intervention, the mean of the correct responses in the pre-test was *M* = *0.55* or 55%, whereas in the post-test, the mean was *M* = *0.87* or 87%, an overall 32% increase. After disseminating the intervention and strictly examining the post-survey scores, most of the women were aware that CMV can spread through urine (93% for both the virtual and in-person groups), can cause hearing loss (91% for the on-line group, and 100% for the in-person group), can cause long-term health complications, including seizures (94% for the on-line group, and 80% for the in-person group), and can be prevented with proper hand hygiene (93% for the virtual group, and 100% for the in-person group). The survey data are summarized in Table 2.

Although most of the women were able to correctly answer most of the statements after the interventions in the respective groups, there were still some misconceptions present. Many of the participants were not aware of how common CMV is (68% post-test incorrect answers for the virtual group, and 27% incorrect answers for the web-based module group), nor were they aware that, in some cases, treatments are available (73% of the subjects in the in-person group correctly responded, post-intervention, that it was not correct that there were no treatments available, and a total of 78% of the respondents got this question correct in the post-intervention web-based module group). For the in-person event, the mean of the number of women who correctly answered each statement in the pre-survey was *M* = *0.65 or* 65%. After the intervention, their mean score changed to M = *0.87* or 87%, an overall 22% increase in knowledge of cCMV. These percentages are cumulative for all women in both groups, including those who indicated that they had (40% in-person group, 44.8% on-line group; Table 1) and those who had not (60% in-person, 55.2% on-line; Table 1) heard about cCMV before signing up for the survey. The results for each individual question in the pre- and post-test questionnaire for each group are shown in Table 2.

We chose to ask subjects in this exploratory study to answer both the pre- and post-test questions about whether or not they had heard of cCMV prior to signing up for this study, our rationale being that every participant had, in fact, been given some information about cCMV prior to the time of study enrollment (see “Methods” section). We next wanted to evaluate whether the fact that a participant indicated pre-survey knowledge of CMV impacted their responses to the invention for both groups. Overall, as noted above, most of the respondents were not familiar with cCMV at the initial time of study recruitment (Table 1). In sum, of the 111 participants in both studies, 56% were unaware of cCMV.

First, we analyzed the effect of pre-survey knowledge about cCMV for those who participated in the web-based survey. Although all participants had been given some information about cCMV at the time of recruitment, of the 96 participants completing the web-based survey, 43 respondents indicated prior awareness of cCMV and 53 reported no prior knowledge. A statistical analysis revealed a significant main effect for familiarity with cCMV at the time of study enrollment on pre-test scores [F(1, 94) = 4.42, p = 0.038]. This indicated that there was a notable difference in the pretest scores between participants who had heard about cCMV in the past, and those who had not. The outcome also demonstrated that approximately 4.5% of the variability in pretest scores of the web-based survey could be attributed to participants’ familiarity with cCMV as measured by R-squared (0.045). The analysis of post-scores also revealed that there was a significant effect of cCMV familiarity on post-test scores [F(1, 94) = 5.127, p = 0.026]. The size of this effect, as measured by R-squared (0.052), suggested that approximately 5.2% of the variability in post-test scores could be attributed to participants’ familiarity with cCMV.

We next examined similar data from the in-person survey, which included 15 participants, with 9 individuals indicating prior awareness of cCMV and 6 reporting no prior knowledge (Tables 1, 2). The analysis of pre-test scores did not reveal a significant main effect of cCMV familiarity to the respondents scores, F(1, 13) = 0.040, p = 0.845. This indicated that there was no substantial difference in the pre-test scores between participants who had heard about cCMV and those who had not in the in-person survey, albeit with a small sample size in this group.

Next, a paired sample *t*-test was employed to measure the mean difference between a set of paired pre- and post-test survey scores for both cohorts combined, to assess the overall impact of the intervention for both groups pooled together. We wanted to gauge whether self-identification of knowledge of cCMV pre-survey had an overall impact on response to the educational intervention. We stated for the null hypothesis, namely, that there would be no difference between participants’ pre- and post- scores, while for the alternative, there would be a significant difference in the pre- and post-scores of each participant. The *t*-test indicated that the web-based intervention was statistically significant (p = 0.001), demonstrating improvement in scores after the web-based intervention was not likely caused by chance alone, but that instead the participants gained knowledge about cCMV from the intervention. The in-person assessment was also statistically significant (p = 0.007), although numbers were small.

In addition to identifying how impactful the intervention was in teaching women about cCMV, the objective of the study was to also assess which modality (the online module, or in-person, face to face educational program and intervention) was more effective. We found that the overall mean difference of the post-test for both cohorts was identical (87% vs. 87%). To further investigate, a multiple linear regression model was employed to further investigate which intervention was more effective; γ = 0.0849χ^1^ + 0.0168χ^2^ + 0.7947, where y is the expected post-test score, χ^1^ is the pre-test score, and χ^2^ is the intervention. When the in-person module was denoted as 1 and the online module denoted as 2, women participating in the in-person event scored a 2% higher score in the post-test evaluation for the online module, (95% CI [− 8%, 12%]), when adjusted for the pre-test score. Thus, there was no evidence that one educational intervention was superior to the other with respect to its ability to confer knowledge to the two groups.

### 3.3 Survey data: impact of age, education, and reproductive history

Next, chi-square tests were performed to assess the relationship between pre-survey familiarity with cCMV and three categorical variables (age, education, and gravidity). The chi-square tests showed no significant relationship between the variables [p = 0.618 (online survey), p = 0.582 (in-person) for age; p = 0.784 (online survey), p = 0.577 (in-person) for education; p = 0.254 (online survey), p = 0.833 (in-person) for gravidity]. Cross-tabulations obtained from the test provide a more visual representation of the data, as demonstrated in Table 3. The majority of respondents in the web-based survey who had heard about cCMV prior to engaging in the study were between 28–34 years (16 subjects; 16/35 or 45.7% in this specific age group, and 16/96 or 17% overall). Though numbers were small, women under 20 years of age were likely to be familiar with cCMV (6 subjects total; 6/11 or 55% in this specific age group; 6/96 or 6% overall). For the in-person survey, the highest percentage of respondents who have heard about cCMV were aged 35 years and above (3/7 in this age group, 43%, and 3/15 [20%] overall). Those younger than 27 years of age were less likely to have heard about cCMV (2/7 in this age group; 29%; 2/15 [13%] overall). However, as noted above, these were not statistically significant differences. Furthermore, we looked at the relationship between educational level and a participant’s familiarity with cCMV. In the web-based survey, we found that the percentages with familiarity with cCMV were similar across all groups. For the in-person session, 2/6 respondents (33%) with an associate degree/some college indicated an awareness of cCMV, while 3/8 respondents (38%) with a high school education or less had heard about cCMV. For one participant in the in-person module, educational attainment information was not available.

In terms of gravidity, it was observed that there was no significant increase in cCMV awareness amongst women who were pregnant before when compared to those who had not been pregnant. This was similar to a previous survey study in Minnesota [34] which, remarkably, showed that recently-pregnant women were no more likely to be aware of CMV than never-pregnant women after adjusting for potential confounders.

There were still some areas for improvement identified in awareness around how common cCMV is, and the therapies available. In response to the statement, *“cCMV is not common”*, 54% of women participating in the web-based intervention correctly selected false in the pre-survey but only 32% correctly selected false in the post-survey, demonstrating, paradoxically, that the educational module enhanced uncertainty about the ubiquity of cCMV. In the in-person session, 60% of the women correctly selected false in the pre-survey statement inquiring about how common cCMV is compared to 73% in the post-survey. Thus, face-to-face education may be more effective in communicating this point. The other statement where more information may need to be provided was regarding availability of treatment. In inquiring about whether it was true that there was no treatment available for cCMV in the web-based survey, 56% of the women in the presurvey correctly chose false in comparison to 78% of the women in the post-survey. In the in-person session, 60% of the women in the pre-survey correctly selected false in comparison to 73% of the women in the post-survey. These both show an improvement in knowledge, but a suboptimal enhancement of knowledge, since antivirals, particularly for symptomatic infants with cCMV, are an important part of clinical management [36]. We did not make the details of antiviral treatment a major focus of the study, since these details were beyond the scope of our survey; rather, the goal was to gauge general awareness among the participants regarding whether they were aware that antiviral agents for CMV were available.

### 3.4 Qualitative data

In the in-person intervention, an open discussion period allowed for collection of comments, questions, and personal experiences shared by participants. Several themes emerged (listed below).

#### 3.4.1 1) Concerns about lack of availability of information

During the discussion of how common cCMV is and how it affects individuals of all ages, race and economic or educational backgrounds, one of the participants brought up a question, “as an educated Somali woman, I am surprised that this is the first time I am hearing about this virus. If this is such a common virus, how come we don’t know much about it?” This question acknowledges the need to increase awareness about CMV, and to identify spaces within the community where women regardless of their background can learn and engage in discussion about cCMV.

#### 3.4.2 2) Perceived susceptibility and 3) ways to mitigate the severity of cCMV

Upon learning about the symptoms associated with cCMV and its potential for producing long-term sequelae for an infant when infection is acquired during early gestation, another participant consulted about a friend who recently emigrated from Somalia who has a child with similar symptoms that was discussed (e.g., microcephaly). This highlighted the impact of providing information about cCMV to the larger community. Other questions that came up during the discussion were about methods to reduce the severity of injury associated with this diagnosis through early detection. One participant inquired about “the best time to detect the virus and the different treatments and modalities available to reduce transmission”. Others asked about symptoms an expectant mother may exhibit which may indicate an infection. These questions again demonstrate the need present within the community to not only learn about cCMV, but also to share their lived experiences and to discuss how they can be engaged in efforts to increase awareness.

## 4 Discussion

While strides in increasing awareness of cCMV have been realized substantially over the past several years, gaps in knowledge remain in communities of color. As cCMV is a disease of health disparities [19], it’s known to have a higher prevalence among racial and ethnic minority populations, similar to COVID-19 [37]. No studies have yet to explore Somali women’s knowledge and beliefs surrounding cCMV. In our web-based and in-person sessions, we found that there are opportunities to provide more education around cCMV. In both sessions, we identified a significant impact that our educational module had on increasing cCMV knowledge and awareness. The study did not find significant difference in awareness of cCMV amongst Somali women prior to the study on basis of their age, education, or pregnancy history. Familiarity with cCMV prior to engaging in the study was similar across age groups and educational background.

It is crucial to consider the specific difficulties faced by the Somali community when examining awareness of cCMV in this group. Language barriers, stigma, and the complex nature of the US healthcare system are some of the challenges that contribute to these difficulties. During the initial phase of raising awareness about the study, there were instances of confusion and uncertainty expressed by members of the community. Some individuals asked if this was a new virus or if it was related to the Monkeypox (now known as M-pox) virus. Minority populations may experience discrimination and structural inequalities, which can lead to a lack of trust in the medical system [38] and poor access to health information [39]. This can further exacerbate existing health disparities, particularly for highly preventable conditions like cCMV. Misinformation may also spread if preventative measures are hindered by such experiences. For example, some parents may hesitate to vaccinate their children due to fears of autism [23–26, 38]. To address these issues, it is essential to build trust early and engage in open discussions about the screening program. Previous traumatic experiences could have an impact on a parent’s views, but trust and engagement can be improved by working to ensure that providers take the time to answer their questions [39].

Additionally, research has shown that inadequate access to information can exacerbate health disparities and decrease incentives for adopting healthy behaviors [39]. During our study, we witnessed this when a participant, who shared her educational background, expressed frustration over why the community is only now learning about cCMV. This highlights the current need and opportunity to educate the community on best practices and on the newborn screening program. Although understanding of CMV infection may be limited, women have a desire for information [35]. Prior studies have found similar results among women who initially had a low awareness of cCMV infection but, after being informed of its prevalence and risks, expressed support for screening themselves and their newborns [34].

In addition to a notable increase in awareness about cCMV among the study participants, we also observed that they were highly eager to learn. Developing a strong relationship with the participants was crucial in enhancing their involvement in both interventions. Several effective strategies were employed to establish trust with the women. The community is an important source of identity for many Somalis and serves as a foundation for shaping their attitudes and beliefs around health information. Family support and encouragement also play a vital role in promoting positive health behaviors [40]. Given the significance of early engagement, we collaborated with community leaders to conduct the research in a culturally sensitive manner and partnered with an Imaam (spiritual leader) to achieve our goals. The educational session was held at a local mosque, and we visited several Friday congregations to introduce ourselves, provide background information about cCMV, and answer any questions the community may have had about our role, intention, and research.

We were surprised by the higher-than-expected level of knowledge about cCMV in our study population. It was surprising that 40–45% of the surveyed group had heard of CMV. In an awareness study by Cannon et al. [41], only 13% of women in a HealthStyles survey had heard of cCMV. We believe that the high level of awareness may be due to some aspects of the study design. In recruitment, in particular for the in-person intervention, some preliminary information was provided to prospective participants. For the in-person survey, verbal recruitment at the participating mosque was used to solicit interest in the study. For the in-person intervention, we also created a simple handout that explained aspects of the study, specifically, that it was focused on cCMV, a common cause of birth injury. Thus, participants had some (minimal) information about cCMV provided in advance. However, a significant limitation of our study is that this point about prior knowledge of cCMV could have been more clearly accounted for. The first question in the questionnaire was: “I have heard about CMV prior to this event”. Considering that all participants receiving some information about congenital CMV at recruitment before answering the questionnaire, this result of this study should be interpreted with caution. It is likely that there was an overestimation of awareness of cCMV in this study, accounted for through our study design and the pre-enrollment information about cCMV given to participants. This probably contributed to an over-estimate of the true level of background knowledge of cCMV in this population. We will be mindful of this limitation in future survey design and implementation studies of cCMV in this and other populations in Minnesota.

Future studies will also consider other potential factors that could have increased awareness of CMV among Somali women in this study, including high parity among women in the study, potential self-selection in recruitment for women who knew about CMV during advertisement for the in-person session (as noted above) and, most notably, work that’s been done in MN to increase knowledge of CMV. Recently, legislation in Minnesota has resulted in a universal screening program for cCMV, and this program also includes resources for community education. Of note, the general level of knowledge and awareness of other important medical issues has been reported to be high in the Somali-American community in Minnesota. For example, Harris et al. reported a survey study of COVID-19 knowledge in Somali, Latinx and Karen residents (average age 37 years; 56.3% female respondents) in which survey participants “consistently had accurate knowledge about COVID-19” and “were attentive to finding trustworthy information” [42].

We also noted that, overall, even in spite of the pre-survey recruitment educational elements, that most of the respondents were not familiar with cCMV (Table 1). Of those who participated in the online survey 53 (55%) were not familiar with cCMV. Of those who participated in the in-person session (n = 15), 9 (60%) were unfamiliar with cCMV. Therefore, although knowledge and awareness were higher than might be expected based on the HealthStyles survey [41] and other studies, we nonetheless observed significant knowledge deficits in this population, which was our prediction going into the study.

Health practitioners are highly regarded in the Somali community and can have an impact in promoting greater understanding of cCMV [43]. Educational content needs to be culturally tailored, insofar as such programs have been reported to be helpful in parental engagement and retention [44]. All in all, we found that collaborating with existing community organizations and providing culturally tailored education were the building blocks to increasing awareness of cCMV in the Somali community. Exercising these efforts may help to promote a wider acceptance of the cCMV screening program and its implications within the Somali community.

The results of the study are encouraging as Minnesota has become the first state to implement universal cCMV screening for newborns. This may lead to increased interest and inquiries about the virus within the Somali community, particularly among parents whose children test positive. To address this, culturally appropriate education must be provided to Somali families by the MDH and care providers. These discussions can play a crucial role in helping families make informed decisions about seeking care for cCMV-related health complications. Previous research has shown that women who received education about CMV were more aware and more likely to adopt recommended hygiene practices to reduce transmission [35]. Studies aimed at promoting awareness have found increased preventive behaviors, such as proper hand washing and safe sexual practices, after educational interventions. This strategy is vital, insofar as cCMV is a common, often symptomless virus that can escape clinical detection and recognition. Moreover, no vaccine is available. Hence, prevention, through behavioral modification, is of paramount importance.

This study had several limitations. There was a variation in the language utilized in the intervention. Both Somali and English were spoken to participants during the face-to-face educational event, while in the online session, only English was offered. Since this was a pilot study meant to gauge the role of our intervention, we had all participants perform a “before and after” summary of knowledge gained, irrespective of whether they self-identified as having any CMV awareness in the pre-intervention questionnaire. Another limitation was that there wasn’t a platform provided to respondents in the online session to engage in a question-and-answer session, as there was in the in-person session. We did not include a mention of the important role of saliva in transmission of CMV infection, and this should be included in future studies of knowledge and awareness of modes of prevention of CMV during pregnancy. Lastly, there was no mechanism to evaluate long-term retention of new knowledge about cCMV. It is hoped that this can be the basis for future studies.

## 5 Conclusions

The main conclusion from our study is that an educational intervention had a significant impact on knowledge and awareness of cCMV in a Somali community. Two survey study strategies (in-person and online), consisting of a pre-survey test, educational intervention, and a post-survey test, were effective in increasing knowledge and perceptions about cCMV among Somali women aged 18 to 40 years old. Awareness of CMV is high in the Somali community compared to the general population. Prior to engaging in the study, 40% of Somali women had heard about CMV in an in-person session conducted whereas 44.8% of Somali women had heard about CMV in a web-based survey. Post-completion of the educational session, there was a substantial increase in knowledge around womens’ awareness of cCMV, the symptoms associated with congenital infection, and in the likely routes of transmission. These results are promising as cCMV is an infection of health disparities.

This information learned in this study can be useful in improving the health equity gaps that exist in this population. Encouragingly, awareness of CMV is already high in the Somali community compared to similar studies previously performed in the general population. Post-completion of the educational session, there was a substantial increase in knowledge around cCMV. This information learned in this study can be useful in improving this health equity gap. The small number of participants in both groups (online and in-person survey) makes it challenging to reduce non-response bias and to compare the efficiency of educational intervention in the pre-and post-test groups, and more studies are needed. Since the Somali community in Minnesota has been affected by vaccine misinformation, leading to preventable disease outbreaks in children, strategies to increase trust and enhance the dissemination of knowledge are of vital importance. As Minnesota adopts universal cCMV screening, future work will be required to ensure that infants with congenital infections in the Somali community undergo appropriate diagnostic evaluation and anticipatory monitoring of neurodevelopment and hearing, to ensure optimal incomes in this high-risk population.

## Figures and Tables

**Table 1:** Participant familiarity with cCMV in the online vs in-person surveys

	Online Survey			In-Person	
	Answer	Frequency	Percent	Frequency	Percentage
I have heard about cCMV	True	43	44.8	6	40
	False	53	55.2	9	60
Total		**96 Participants**	**100%**	**15 Participants**	**100%**

**Table 2:** Pre- and post-intervention cCMV awareness survey scores for study participants

Survey statements	Web-based module (96 subjects)		*P* value	In-person module (15 subjects)		*P* value
	Pre-test % with correct answer	Post-test % with correct answer		Pre-test % with correct answer	Post-test % with correct answer	
1) CMV is not common (correct answer: FALSE)	52 (54%)	31 (32%)	0.002	9 (60%)	11 (73%)	0.43
2) CMV is preventable (correct answer: TRUE)	59 (61%)	91 (95%)	< 0.001	11 (73%)	14 (93%)	0.189
3) CMV can spread through urine (correct answer: TRUE)	40 (42%)	89 (93%)	< 0.001	9 (60%)	14 (93%)	0.02
4) CMV can spread through hugging or cuddling (correct answer: FALSE)	62 (65%)	88 (92%)	< 0.001	8 (53%)	12 (80%)	0.04
5) CMV can cause hearing loss in a newborn baby (correct answer: TRUE)	51 (53%)	87 (91%)	< 0.001	11 (73%)	15 (100%)	0.04
6) CMV can cause long-term health problems, such as seizures (correct answer: TRUE)	63 (66%)	90 (94%)	< 0.001	10 (66%)	12 (80%)	0.43
7) Washing your hands stops the spread of CMV (correct answer: TRUE)	51 (53%)	89 (93%)	< 0.001	11 (73%)	15 (100%)	0.04
8) There are no treatments available for CMV (correct answer: FALSE)	54 (56%)	75 (78%)	0.001	9 (60%)	11 (73%)	0.43

**Table 3:** Examination of familiarity with cCMV based on response to query: “I have heard about CMV”

Statement: I have heard about cCMV			
		True	
		Count (% of total)	
		Online survey	In-person
Total		43/96 (44.8%)	6/15 (40%)
Age	Under 20	6/11 (54.5%)	2/5 (40%)
	21–27 years^a^	9/26 (34.6%)	0/2 (0%)
	28–34 years	16/35 (45.7%)	1/1 (100%)
	35 years and over	12/24 (50%)	3/7 (42.9%)
Education	Associate degree/some college	14/30 (46.7%)	1/4 (25%)
	College degree or more	11/28 (39.3%)	1/2 (50%)
	High school or less	18/38 (47.4%)	3/8 (37.5%)
	Unknown^b^	0	1
Gravida	Yes	30/61 (49.2%)	3/7 (42.9%)
	No	13/35 (37.1%)	3/7 (42.9%)
	Unknown	0	1
Household Income	Less than $19,000	5/10 (50%)	1/1 (100%)
	$20,000–$49,000	12/34 (35.3%)	1/5 (20%)
	$50,000–$99,000	20/40 (50%)	1/4 (25%)
	$100,000 or more	6/12 (50%)	2/4 (50%)
	Unknown^b^	0	1

Online and in-person survey data for participant’s age, education level, and gravidity

a For the age group (21–27 years), 0/2 implies that there were two respondents under the category but neither of them was familiar with cCMV

b In the unknown rows, (1) represents a missing response while (0) represents no missing response

## Data Availability

Data from this study are stored in a secured site maintained by the UMN Center of Excellence for HIPAA Data (https://it.umn.edu/services-technologies/box-secure-storage; accessed on 11 August 2023). Data are available on request.

## Abbreviations

cCMV: Congenital Cytomegalovirus
CHIMES: CMV and Hearing Multicenter Screening
CMV: Cytomegalovirus
IRB: Institutional Review Board
MDH: Minnesota Department of Health
MMR: Mumps-Measles-Rubella
SPSS: Statistical Product and Service Solutions
UMN: University of Minnesota
